# Design of Wideband GHz Electric Field Sensor Integrated with Optical Fiber Transmission Link for Electromagnetic Pulse Signal Measurement

**DOI:** 10.3390/s18093167

**Published:** 2018-09-19

**Authors:** Guogang Zhang, Wenfan Li, Lu Qi, Jingcun Liu, Zhengxiang Song, Jianhua Wang

**Affiliations:** State Key Lab of Electrical Insulation and Power Equipment, Xi’an Jiaotong University, No. 28 Xianning West Road, Xi’an 710049, China; 18109314169@163.com (W.L.); eastlife0926@foxmail.com (L.Q.); liujingcun0523@163.com (J.L.); zxsong@mail.xjtu.edu.cn (Z.S.); jhwang@mail.xjtu.edu.cn (J.W.)

**Keywords:** EMP measurement, E-Field sensor, optical fiber transmission link, D-dot antenna, transient overvoltage, partial discharge

## Abstract

The detection of high frequency overvoltage and partial discharge is of great significance in evaluating the insulation condition of high-voltage power equipment. A wideband GHz electric field (E-field) sensor for electromagnetic pulse (EMP) signal measurement was proposed in this paper. An optical fiber transmission link was adopted in the design in order to implement high-voltage isolation and reduce electromagnetic interference during transmission. The designed sensor was mainly made up of a differential electric field (D-dot) antenna, transmitter, and receiver. The D-dot antenna was designed to detect high frequency E-field strength and generate a corresponding voltage signal, which was converted into an optical signal by the transmitter. The optical signal could be transmitted a large distance through an optical fiber without electromagnetic interference and changed back to a voltage signal again by a receiver. The design process of the sensor was introduced in detail, and experiments were performed using a gigahertz transverse electromagnetic (GTEM) cell and an EMP simulator to verify it. The results indicated that the designed sensor had a good performance besides an expectable delay due to the optional amplifier.

## 1. Introduction

Electromagnetic pulse (EMP) measurement is of great significance in many scientific and technical fields, such as electrostatic discharge, lighting, high-power microwave (HPM), high-altitude nuclear electromagnetic pulse (HEMP), very fast transient overvoltage (VFTO), and partial discharge (PD) in high voltage engineering fields, electric field (E-field) monitoring in medical apparatuses, and ballistic control [1,2]. Common problems existing in power equipment, such as high-frequency transient overvoltage and partial discharge, seriously endanger the safety of power systems, and many innovative approaches have been proposed from the laboratory scale to practical application [3]. EMP measurement can be applied to help judge the insulation status of power equipment by detecting and analyzing the high frequency E-field induced by transient overvoltage or partial discharge in high-voltage power equipment [4,5]. There has been rapidly increasing interest in EMP measurement during the last few decades [6,7]. Generally, EMP sensors should have a large intrinsic bandwidth of several GHz, fast response time (usually about a nanosecond), a flat frequency response, and a detectable range within several kilovolts per meter to tens of kilovolts per meter [8].

Si presented a design and testing of a small E-field antenna to measure a lightning electro-magnetic pulse (LEMP), but the sensor was only designed for lightning measurements. It cannot measure weak electromagnetic pulses and its designed bandwidth is less than 150 MHz [9]. Kong presented a norm detector without an external connecting line for transient electrical signal measurement based on high-speed digital acquisition and storage technology that can measure the coupled electrical signals in hermetically-sealed cavities and calculate the commonly used norms of the measured signal using a specially designed signal processing software [10]. Azpúrua presented a digital measurement system intended to be used to assess the radiated electromagnetic interference (EMI) in both time and frequency domains in his paper [11]. Wang’s study illustrated that the existing common path interferometer (CPI)-based integrated optical E-field sensors (IOESs) could not meet the sensitivity requirements for some of the applications of intense E-field measurement. In his research, the sensitivity of integrated optical E-field sensors based on common path interferometer was improved by designing dipole antennas and electrodes around the waveguide [12]. In Yang’s work, an integrated optical E-field sensor with segmented electrodes using a LiNbO_3_ Mach-Zehnder optical modulator is designed and fabricated [13]. Drexler gave the overview of several methods suitable for the measurement of short solitary pulses with a high power level [14]. Although an electro-optical crystal-sensing method has advantages in reducing the EMI and minimizing the perturbation to the measured electromagnetic field, the availability of the special sensing element and the cost of additional optical measurement systems still block the wide application of this kind of sensors.

In short, most studies focused on optimizing the measurement antenna and seldom mentioned the transmission problem of the measurement result. The common EMP sensors use a coaxial cable to transmit the measurement results of the antenna. However, the coaxial cable link has certain limitations. It is vulnerable to electromagnetic interference and the transmission distance is limited considering the degradation of a high-frequency signal. An optical fiber link has the advantages of a wider bandwidth, lower loss, farther transmission distance, and stronger immunity from EMI than coaxial cables, so we applied it to the transmission of the EMP signal. In this article, a wideband GHz electrical field sensor integrated with an optical fiber transmission link was designed. We utilized a differential electric field (D-dot) antenna to measure the electric field and an optical fiber link to transmit the analog waveform in real time. An amplifier was also designed in our sensor, which can be conveniently connected to the front end of the optical fiber transmission link when measuring weak electromagnetic pulse signals. The D-dot antenna was designed to detect the pulse electrical field strength and generate a corresponding voltage signal. The fiber transmission link was developed to transmit the voltage signal, which was made up of a radio frequency (RF) wideband low noise amplifier (LNA), electro-optic conversion circuit, and photoelectric conversion circuit. The output of the D-dot antenna was amplified using an RF wideband LNA to produce the modulation signal for a laser diode (LD). An electro-optic conversion circuit modulated the LD with the modulation signal to change the electric signal to an optical signal. After the optical signal was transmitted to a faraway receiver through an optical fiber, the photoelectric conversion circuit was used to detect the optical signal and change it into a voltage signal. Design of the sensor is described in detail in Section 2 and experimental verifications were carried out on a gigahertz transverse electromagnetic (GTEM) cell and an EMP simulator in Section 3. The results show that the designed sensor has a good performance. Section 4 summarizes the article and draws the conclusion.

## 2. Design of the Sensor

**Figure 1:**
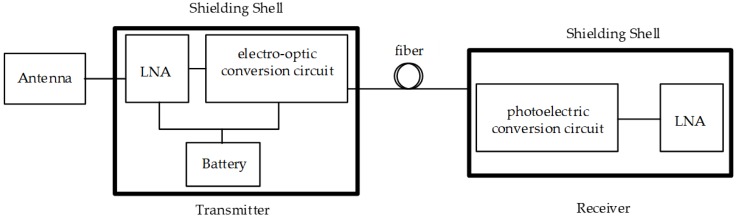
The simplified diagram of the sensor.

The sensor designed in this paper includes two main parts, a D-dot antenna and a fiber transmission link, which is composed of transmitter, optical fiber, and receiver, as shown in Figure 1. To achieve measurements of high accuracy under an electromagnetic environment, the frequency response of the receiving sensor needed to be wide enough to satisfy the bandwidth limit. Furthermore, good linearity, high sensitivity, and large fidelity were required to ensure short-pulse measurements. A wideband GHz D-dot antenna was designed to detect the electrical field strength with a nanosecond rise time and generate the corresponding voltage signal. A fiber transmission link was used to transmit the measurement results a large distance away. The transmitter, which consisted of an RF wideband LNA, an electro-optic conversion circuit, and a rechargeable battery as a power supply, converted the electrical signal into an optical signal. The receiver consisted of a photoelectric conversion circuit and an LNA, the same as that in the transmitter, which restored the optical signal transmitted through the optical fiber to an electrical signal. Shielding shells were used for the transmitter and receiver to prevent those circuits from EMI.

### 2.1. Design of the D-dot Antenna

#### 2.1.1. Calculation for the Shape

**Figure 2:**
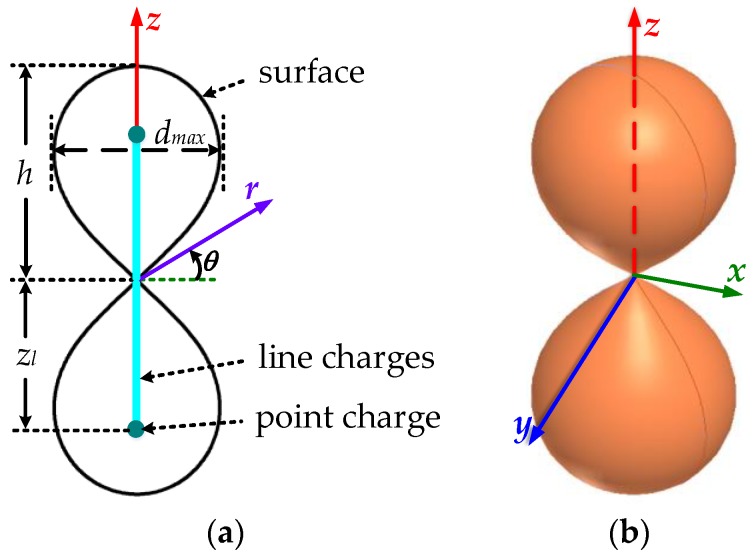
Schematic diagram of the D-dot antenna with (**a**) a cylindrical coordinate system and (**b**) a Cartesian coordinate system.

Based on the asymptotic conical dipole (ACD) antenna model, the basic geometry of the D-dot antenna used in the design could be constructed using the equivalent-charge method shown in Figure 2, whose surface was determined by the potential distribution of line charges and point charges in mirror symmetry [15,16,17,18,19]. Additionally, *h* is the height of the half dipole; $z_{l}$ is a length constant, referring to line charge length, and *d_max_* is the maximum diameter in the landscape orientation.

The antenna shape in the (r,z) coordinates can be obtained by Equation (1), which is a non-linear equation and can be solved using an iterative method [18]:

(1) $$\ln\left(\Theta_{0}^{-2}\right)=\ln\left\{\frac{{\left[z+\sqrt{z^{2}+r^{2}}\right]}^{2}}{\left[z+z_{l}+\sqrt{{\left(z+z_{l}\right)}^{2}+r^{2}}\right]\left[z-z_{l}+\sqrt{{\left(z-z_{l}\right)}^{2}+r^{2}}\right]}\right\}+\frac{\alpha z_{l}}{\sqrt{{\left(z-z_{l}\right)}^{2}+r^{2}}}-\frac{\alpha z_{l}}{\sqrt{{\left(z+z_{l}\right)}^{2}+r^{2}}}$$

where α is the ratio of the point charge to the line charge; $z_{l}$ can be calculated for the case r=0,z=h; and $\Theta_{0}$ is a constant determined by the desired asymptotic impedance and can be deduced using:

(2) $$\ln\left(\Theta_{0}^{-1}\right)=\pi \frac{Z_{c}}{Z_{0}}$$

where $Z_{c}$ is the characteristic impedance of the antenna, $Z_{0}=\sqrt{\mu_{0}/\varepsilon_{0}}$ is the free space wave impedance and $\mu_{0}$ is the permeability of vacuum, and $\varepsilon_{0}$ is the permittivity of vacuum.

**Figure 3:**
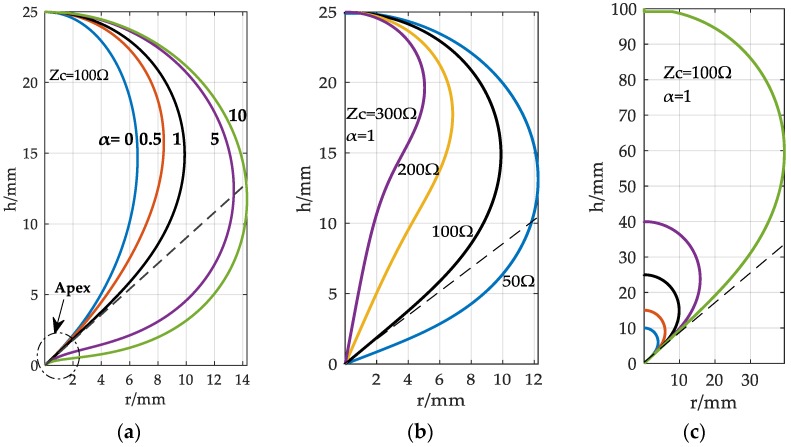
The shapes of the D-dot antenna for various (**a**) α, (**b**) *Z_c_*, and (**c**) *h*.

In order to get the optimal shape of the antenna, various characteristic parameters of a D-dot antenna have been investigated. In Figure 3, shapes of the antenna for variables like α, *Z_c_*, and *h* are drawn typically. Considering that the outlines of the D-dot antenna need to be made more asymptotic, α=1 was confirmed as the optimum value because of the lowest rate-of-change of the slope near the apex, which is shown in Figure 3a. From Figure 3b, a larger *Z_c_* means the apex of the shape becomes more poignant, meanwhile the *d_max_* is smaller. For a dipole antenna, *Z_c_* was designed to be 100 Ω to satisfy impedance matching in a practical application. Figure 3c shows the relationship between antenna shapes and heights under the condition of a certain α and *Z_c_*.

The capacitance between two surfaces of the D-dot antenna is [18]:

(3) $$C_{a}=\frac{2\pi \varepsilon_{0}z_{l}}{\ln\left(\Theta_{0}^{-1}\right)}$$

The antenna equivalent area can be given by [18]:

(4) $$A_{e}=\frac{3\pi z_{l}^{2}}{\ln\left(\Theta_{0}^{-1}\right)}=\frac{3z_{l}}{2\varepsilon_{0}}C_{a}$$

The EMP signal in the form of a differential mode is received by the designed antenna, and it can be regarded as a current source [18]:

(5) $$i=\varepsilon_{0}A_{e}\frac{𝜕E}{𝜕t}$$

**Figure 4:**
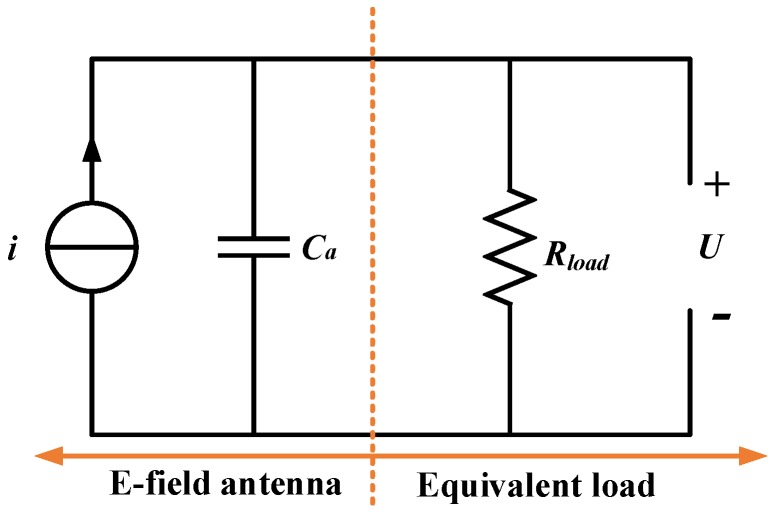
The equivalent circuit of the D-dot antenna.

In Equation (5), *E* is the electric field intensity being measured. The equivalent circuit of the D-dot antenna is shown in Figure 4, where $R_{load}$ is just the matching impedance.

Then, the transform function of the equivalent circuit can be computed in the complex frequency domain by using a Laplace Transform:

(6) $$\frac{U\left(s\right)}{E\left(s\right)}=\frac{s\varepsilon_{0}A_{e}R_{load}}{1+sR_{load}C_{a}}$$

where U is the voltage on the load. The upper frequency of a D-dot antenna can be obtained using:

(7) $$f_{c}=\frac{1}{2\pi R_{load}C_{a}}$$

**Figure 5:**
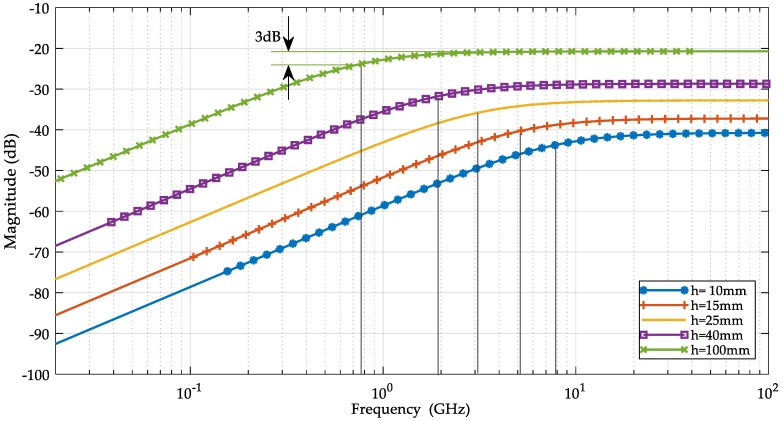
Frequency response of the antennas with different height *h*.

From Equations (1)–(7), the main characteristic parameters can be calculated, given in Table 1, in cases of different heights. The upper frequency $f_{c}$ is related to the assumed *h*, as shown in Table 1 and Figure 5. From the values of *h* and *d_max_*, it can be seen that a smaller size means a larger upper frequency or wider bandwidth. Hence, considering the demand of a nanosecond-fast rise time pulse measurement and miniaturization of volume as a whole, h=25 mm was chosen in this paper, in which case the upper frequency was about 3 GHz theoretically.

**Figure 6:**
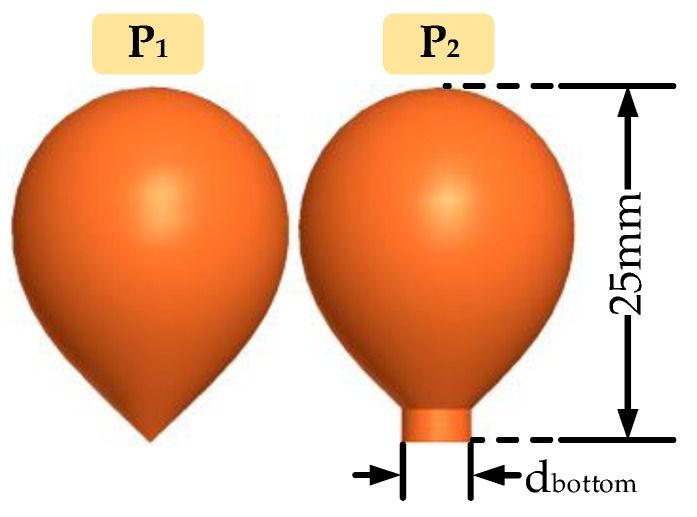
The standard shape (P_1_) and optimized shape (P_2_) of the D-dot antenna (only half is presented because of the symmetrical structure).

In Figure 6, the geometry of the standard-shape antenna (P_1_) can now be defined. Like an upside-down water-drop, the bottom of the asymptotic conical antenna is acute and sharp. Therefore, it is hard to fabricate and easy to fall off from the pedestal in the practical application. To solve these problems, the bottom of our antenna was optimized into a cylindrical shape, as shown in Figure 6 (P_2_). Different diameters of the bottom cylinder were chosen for simulation to compare and find the best one in the following frequency domain analysis part.

#### 2.1.2. Frequency Domain Analysis

**Figure 7:**
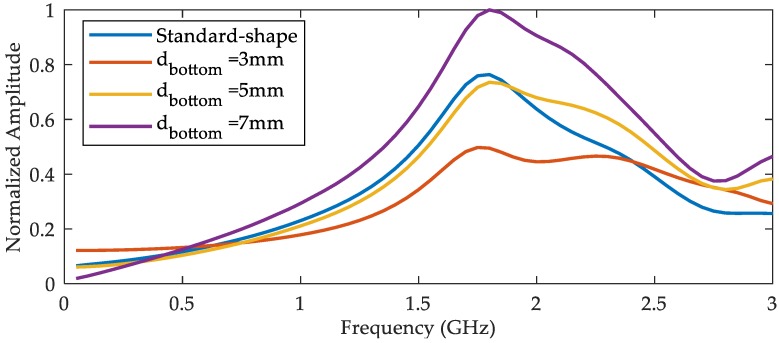
Frequency sweep results from simulation.

In order to find the best bandwidth, the standard-shape antenna and optimized-shape antennas with different diameters of bottom the cylinder are simulated and contrasted in the frequency domain. The frequency sweep responses from simulation are shown in Figure 7. It can be seen that the output signal of optimized-shape antenna was very close to the standard-shape antenna when the diameter of bottom cylinder was 5 mm, so we chose that as the optimum in our design. The optimized antenna in the following refers to the optimized-shape antenna with a 5 mm bottom diameter.

**Figure 8:**
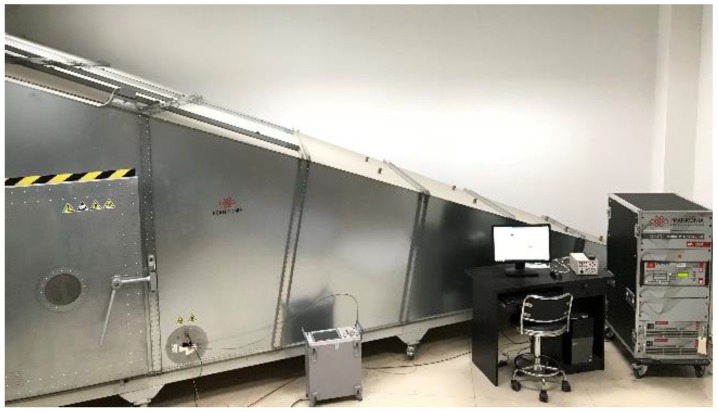
GTEM cell setup for the frequency test.

Experiments under a series of different frequencies were carried out. The experimental setup was a GTEM cell with an excitation source that could modulate frequencies and the field intensity of the electromagnetic field, as shown in Figure 8. Antennas were put into the valid center zone of the cell, where the E-field vector was vertical and the electromagnetic wave could be considered linearly polarized. A R&S (Rohde & Schwarz) ESR EMI test receiver was connected to the antennas to record the output signals through a 50 Ω coaxial cable.

**Figure 9:**
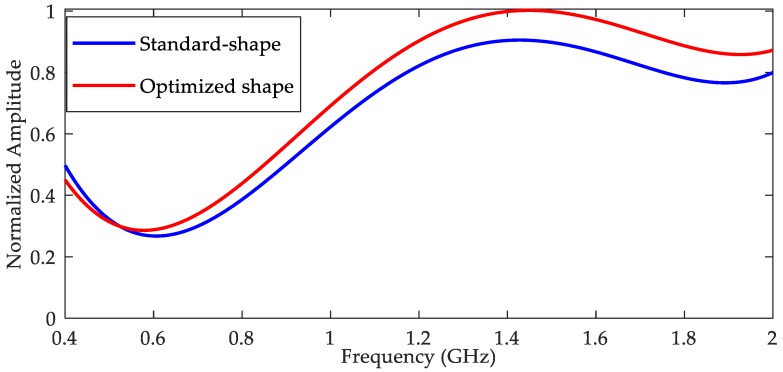
Frequency sweep results for the GTEM cell.

Figure 9 shows a normalized frequency sweep (400 MHz to 2 GHz, step size is 100 MHz) results of the antennas. It can be seen that the output signal of the optimized-shape antenna was very close to the standard-shape antenna when the frequency was under 2 GHz, indicating that the optimized antenna had good performance while solving the problems of processing and installation difficulties.

### 2.2. Design of the Fiber Transmission Link

**Figure 10:**
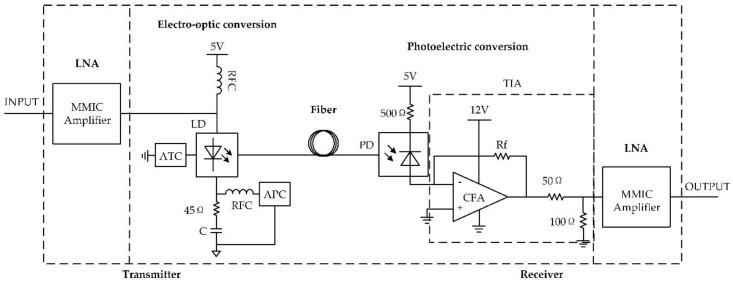
Circuit diagram of the optical fiber transmission link.

As mentioned above, the designed fiber transmission link was composed of a transmitter, optical fiber, and receiver. Its simplified circuit diagram is shown in Figure 10.

The transmitter mainly consisted of two parts: an LNA and an electro-optical conversion circuit. Its main function is to change the input electrical signal into an optical signal that can be transmitted using optical fiber through LD modulation technology. The method of the LD direct modulation was adopted to change the electric signal to an optical signal in this work. There were many studies on LNAs and some achievements had been made [20,21,22]. However, with the rapid development of semiconductor materials and processes, monolithic microwave integrated circuits (MMICs) are becoming ever more popular. MMICs have the advantages of small size, superior performance, high bandwidth, and easy impedance matching, and are now widely used in engineering design [23]. In order to obtain a modulation signal, we selected an InGaP heterojunction bipolar transistor (HBT) MMIC chip and designed a wideband LNA to amplify the output signal of the D-dot antenna and generate a modulation signal for the electro-optic conversion circuit. The designed LNA was unconditionally stable and had a gain of 20 dB and a bandwidth of 4 GHz. Its noise figure was within 3.5 dB, which can fully meet the requirements.

In order to change the electric signal to an optical signal for fiber transmission, an electro-optical conversion circuit was designed. LDs not only have a high degree of monochromaticity, coherence, directionality, and brightness, like general lasers, but also have advantages such as a small size, light weight, direct modulation, and low price. Therefore, they have been more and more widely used in scientific research, medicine, optical communications, and other fields [24,25]. A single-mode distributed feedback (DFB) continuous wave (CW) LD was selected in this paper, which had a center wavelength of 1550 nm. The rise/fall time was 0.3 ns, therefore the bandwidth of this LD was about 1.17 GHz. As the resistance of the selected LD was about 10 Ω, a 40 Ω resistance R was used to match the LD’s impedance to 50 Ω. LD is a kind of current drive device, and the operating life and work characteristics depend heavily on the performance of the drive power supply. The performance and security requirements of the drive power are highly demanding, therefore, to design a drive power fit for the LD technical requirements with a stable performance and reliable operation is quite necessary [26]. The LD output wavelength not only increases with the drive current increasing, but also increases with the temperature increasing. The fluctuations of temperature and drive current would also affect the LD output optical power. An average power controller (APC) that had high stability and low ripple was designed to detect and adjust the LD output power. It included an anti-surge network, filter circuit, and an over-current and over-heating protection circuit to ensure the system’s security and stability. A high-accuracy automatic temperature controller (ATC) was also designed to detect and adjust the LD temperature [27].

The receiver consisted of a photoelectric conversion circuit and an LNA. After the optical signal was transmitted through the optical fiber to the receiver, it needed to be changed back into an electric signal. A photoelectric conversion circuit accomplished the conversion from an optical signal to an electrical signal while the LNA amplified it so that the output voltage could be observed and recorded by an oscilloscope directly. The photoelectric conversion circuit designed in this paper included two parts: an optical detection device and a transimpedance amplifier (TIA). The output impedance of the photoelectric conversion circuit was matched to 50 Ω using two resistances R3 and R4. An InGaAs positive intrinsic-negative (PIN) photodiode was adopted as the optical detection device, which had high dependability, high sensitivity, low capacitance, and low dark current. Its dark current was 30 pA and capacitance was 1 pF. Its −3 dB bandwidth was 2.5 GHz. It could receive an optical signal whose wavelength was from 800 nm to 1700 nm. Considering some requirements, such as high gain, low figures of noise, and wide bandwidth, TIAs are widely used in high-speed optical communication systems [28,29,30]. A PIN photodiode detected the optical signal and generated the corresponding current signal, which should be changed into a voltage signal for signal processing. The function of the TIA was to change a current signal to a voltage signal. A high-performing current feedback amplifier (CFA) was adopted to implement the TIA, and its bandwidth could be theoretically configured to about 2 GHz using a 550 Ω feedback resistor according to the datasheet of the selected CFA. Considering the influence of parasitic parameters, such as the printed circuit board (PCB) layout and component packaging, the actual bandwidth was generally lower than the theoretical expectation, therefore the expected bandwidth of the TIA was about 1 GHz. The photocurrent generated by the photodiode by receiving the optical signal was usually only a few milliamperes, therefore the output voltage of the TIA was correspondingly small. The same type of LNA was connected to the output of the TIA so that the output voltage of the optical receiver could be directly observed on an oscilloscope. The output voltage of the sensor was about tens of millivolts to several hundred millivolts after using the LNA and the voltage could be easily observed and recorded using an oscilloscope.

Fiber transmission has the advantages of a wider bandwidth, lower loss, farther transmission distance, and stronger EMI resistance than the coaxial cable [20], so we used optical fiber as the transmission medium. The optical fiber we used in the fiber transmission link was single-mode. In the laboratory environment, we used a fiber length of about 1.5 m, in which the transmission delay could be omitted in comparison to the delay of the circuit.

## 3. Experimental Verification of the Sensor

**Figure 11:**
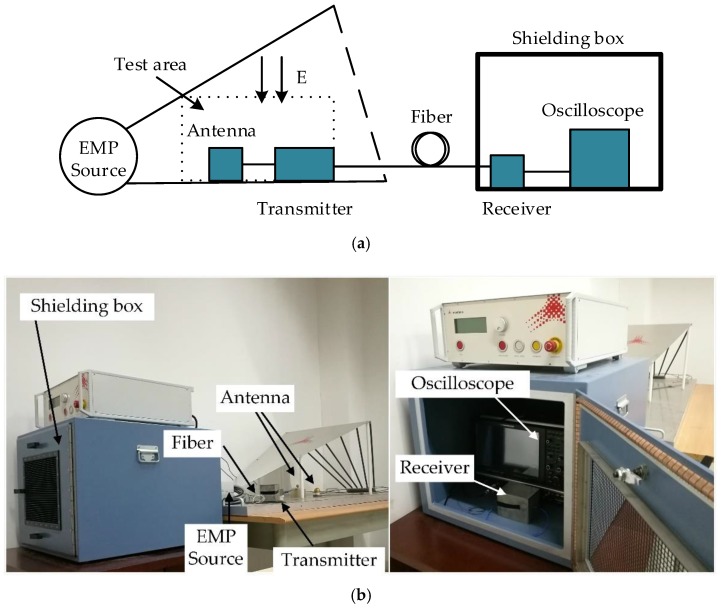
Experiment test platform: (**a**) the schematic diagram and (**b**) the experimental system.

In the sensor design section, performance parameters of the D-dot antenna were optimized and verified in a GTEM cell. In this section, the designed sensor was verified through an EMP simulator (Montena EMP simulator according to MIL-STD-461 RS105) in the time domain. The structure diagram of test platform and the photo of the experimental system are shown in Figure 11a,b, respectively. 

The experimental system was mainly composed of an EMP generator, a standard antenna from the Montena company (Switzerland), a shielding box, and a high-frequency oscilloscope. The EMP generator could produce a single electromagnetic pulse with a 10–90% rise time of 2.3 ± 0.5 ns and a peak field strength of up to 50 kV/m in the vertical direction. According to the analysis above, the signal received by the D-dot antenna was a current differential signal. In this experiment, a passive integrator was used to restore the differential signal to a voltage signal. Since the oscilloscope was susceptible to interference from the electromagnetic environment, the metal shielding box was used to isolate external interference and obtain a more ideal waveform signal. In addition, to avoid the reflection distortion during the transmission of the current signal, each input and output link was matched with the characteristic impedance of 50 Ω.

**Figure 12:**
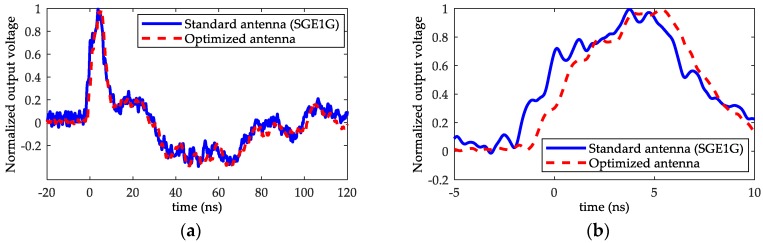
Comparison of output voltage waveforms: (**a**) full waveforms and (**b**) expanded waveforms.

In order to verify the performance of the optimized-shape antenna, placing a standard antenna from the Montena company (SGE1 G) and the optimized-shape antenna in the chamber to detect the EMP signal, the output voltage waveforms of the standard antenna and optimized-shape antenna were recorded and normalized to analyze and compare their performance. Figure 12 shows the test results.

It can be seen that output of the designed antenna was mostly the same as the output of the standard one, which shows that the designed antenna could measure the EMP signal effectively.

Similar to the performance verification experiment of the optimized antenna, the output waveforms of the standard antenna and the designed sensor were recorded and normalized to verify the performance of the sensor with optical fiber transmission link.

**Figure 13:**
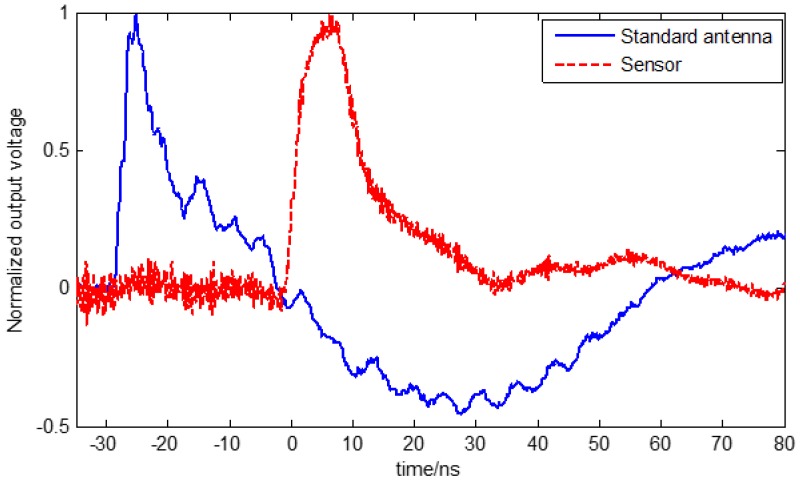
The test results of the standard antenna and the sensor with an optical fiber link.

Figure 13 shows the test results of the commercial standard antenna and the designed sensor with optical fiber transmission link using an EMP simulator. It can be seen that the output waveform of the sensor with the optical fiber link was close to that of the standard antenna, which indicates that the fiber communication system could transmit the measurement result with a transmission delay. The total delay time was about 28 ns, which was mainly due to the LNA circuit and could be significantly eliminated when signal amplification is not required.

## 4. Conclusions

A wideband GHz E-field sensor integrated with an optical fiber transmission link was designed for EMP signal measurement in this paper. The sensor was made up of two main parts: the D-dot antenna and the fiber transmission link. A novel optimized-shape D-dot antenna was proposed to improve its robustness in practical applications and a wideband analog fiber transmission link was designed to transmit the measurement results without electromagnetic interference. The expected bandwidth of the sensor exceeded 1 GHz and it could measure weak electromagnetic pulse signals by connecting the designed amplifier to the front end of the optical fiber transmission link. The results of this article can be summarized as follows:

(1) The optimized D-dot antenna with a 5 mm bottom diameter was easy to fabricate and fix. The structure improved its robustness in practical applications without an evident decline in performance with respect to the standard shape. The bandwidth of the antenna reached about 2 GHz.

(2) The designed fiber transmission had the advantages of a wider bandwidth, lower loss, farther transmission distance, and stronger immunity from EMI than coaxial cables, and could transmit EMP measurement results to the distant location. The DFB laser diode with a center wavelength of 1550 nm and the InGaAs PIN photodiode with a wavelength from 800 nm to 1700 nm were adopted to provide an emitter and receiver, and their bandwidths were estimated to be 1.17 GHz and 2.5 GHz, respectively.

(3) Considering the bandwidths of the main components, including the D-dot antenna, the transmitter, and the receiver, the sensor’s designed bandwidth exceeded 1 GHz. The sensor was verified using the EMP simulator. The experimental results show that the sensor could correctly measure and transmit the electromagnetic pulse with a certain time delay of about 28 ns, which was mainly due to the LNA circuit, through comparison with the commercial standard D-dot antenna. The delay could be significantly eliminated when signal amplification was not required.

## Figures and Tables

**Table 1:** Main parameters comparison of the ACDs.

*h* (mm)	$d_{max}\;(\mathrm{mm})$	$z_{l}\;(\mathrm{mm})$	$C_{a}\;(\mathrm{pF})$	$A_{e}\;({\mathrm{cm}}^{2})$	$f_{c}\;(\mathrm{GHz})$
10	7.91	6.12	0.41	4.24	7.79
15	11.87	9.18	0.61	9.53	5.19
25	19.78	15.30	1.02	26.47	3.12
40	31.64	24.48	1.63	67.77	1.95
100	79.10	61.19	4.09	423.48	0.78
